# Hospital Floors

**Published:** 1902-07-19

**Authors:** Sydney Holland


### Editor's Letter-Box.

[Our Correspondents are reminded that prolixity is a great bar to publication, and that brevity of style and conciseness of statement greatly facilitate early insertion.]

HOSPITAL FLOORS.

Dear Sir,—I have had experience during the last twelve years of the following materials for floors—deal boards, teak boards, terrazo mosaic, linoleum on cheap deal boards, and I am quite sure that the latter, if good linoleum is used and it be well laid, is the best. It is the easiest and cheapest to clean, the most pleasant to walk on, less tiring, less noisy, and for patients warmer than mosaic and less slippery than polished boards. And it is the cheapest.—Yours,

Sydney Holland.

P.S.—Some people may fear that linoleum is absorbent, but Dr. Bullock, our bacteriologist, has been lately testing the linoleum we have had down in the London Hospital operating rooms during the last two years—5,000 operations—and can discover no germs of any sort.

